# Motor Performance But Neither Motor Learning Nor Motor Consolidation Are Impaired in Chronic Cerebellar Stroke Patients

**DOI:** 10.1007/s12311-019-01097-3

**Published:** 2020-01-30

**Authors:** Franz Hermsdorf, Christopher Fricke, Anika Stockert, Joseph Classen, Jost-Julian Rumpf

**Affiliations:** Department of Neurology, University of Leipzig, Liebigstr. 20, 04103 Leipzig, Germany

**Keywords:** Motor learning, Motor consolidation, Motor control, Cerebellum, Stroke

## Abstract

The capacity to acquire and retain new motor skills is essential for everyday behavior and a prerequisite to regain functional independence following impairments of motor function caused by brain damage, e.g., ischemic stroke. Learning a new motor skill requires repeated skill practice and passes through different online and offline learning stages that are mediated by specific dynamic interactions between distributed brain regions including the cerebellum. Motor sequence learning is an extensively studied paradigm of motor skill learning, yet the role of the cerebellum during online and offline stages remains controversial. Here, we studied patients with chronic cerebellar stroke and healthy control participants to further elucidate the role of the cerebellum during acquisition and consolidation of sequential motor skills. Motor learning was assessed by an ecologically valid explicit sequential finger tapping paradigm and retested after an interval of 8 h to assess consolidation. Compared to healthy controls, chronic cerebellar stroke patients displayed significantly lower motor sequence performance independent of whether the ipsilesional or contralesional hand was used for task execution. However, the ability to improve performance during training (i.e., online learning) and to consolidate training-induced skill formation was similar in patients and controls. Findings point to an essential role of the cerebellum in motor sequence production that cannot be compensated, while its role in online and offline motor sequence learning seems to be either negligible or amenable to compensatory mechanisms. This further suggests that residual functional impairments caused by cerebellar stroke may be mitigated even months later by additional skill training.

## Introduction

The ability to acquire and retain motor skills is a complex phenomenon and a key prerequisite for successful everyday behavior and functional independence across the lifespan. Moreover, this capacity is particularly relevant for recovery from compromised motor skills due to brain damage such as stroke. Learning a new motor skill as well as reestablishing an impaired or lost motor skill due to brain damage relies on repeated practice and passes through distinct learning stages [1, 2]. Much of the knowledge about these dynamic processes has been gained by investigating motor sequence learning—an extensively used and ecologically valid paradigm that tests the ability to integrate single elements of a movement into a coherent and smoothly performed unit (e.g., [3, 4]). When practicing a sequential motor skill for the first time, performance typically improves quickly within a training session as a function of practice referred to as fast online learning. Following the fast online learning phase, the initially labile training-induced motor memory is transformed into a more robust representation in the absence of further skill practice. This offline phase is referred to as consolidation, defined as resistance against interference (i.e., stabilization) or performance increments between the end of a training session and retesting (i.e., offline learning) [5, 6].

Previous research has demonstrated that online and offline phases of explicit sequential motor skill acquisition are mediated by specific dynamic interactions between nodes of a wide-spread neural network that encompasses the primary motor cortex, basal ganglia, supplementary motor area, hippocampus, and the cerebellum [2, 7–11]. Despite extensive research, the role of the cerebellum during online and offline motor sequence learning remains controversial. Theoretical models derived from imaging studies propose that the cerebellum is mainly recruited during the early online learning phase of motor sequence learning and that its activity diminishes as a function of practice to become untraceable when the practiced skill is well-established and training-induced performance increments level off [3, 5, 9, 12, 13]. Further evidence on the role of the cerebellum in motor learning has been obtained by non-invasive brain stimulation studies which, however, provided mixed results. Some of them reported bidirectional modulation of online motor learning in the way that training-induced performance increments were improved after “inhibitory” priming of the cerebellum, while “facilitatory” cerebellar priming impaired online motor sequence learning [14, 15]. Others, however, reported opposite results suggesting that “facilitatory” cerebellar stimulation improved online motor sequence learning [16, 17], whereas training under the influence of “inhibitory” cerebellar stimulation led to an impairment of online performance gains [18]. Results were similarly inconsistent when the question was investigated whether the cerebellum plays a relevant role for consolidation of training-induced skill formation. While Wessel and colleagues [19] as well as Samaei and colleagues [20] observed improved offline consolidation following motor sequence training combined with “facilitatory” anodal cerebellar transcranial direct current stimulation (in the absence of an effect on online learning), Cantarero and colleagues [16] reported no effect on consolidation (but improved online learning).

Another open question with respect to the role of the cerebellum during motor sequence learning is whether lateralized motor sequence learning functions in the brain may have corresponding lateralized counterparts in the cerebellum. Chronic unilateral lesions of the cerebellum due to stroke were shown to impair training-induced performance increments in an implicit motor sequence learning task when the task was performed with the ipsilesional hand, while learning with the contralesional hand remained unaffected [21]. The majority of motor learning studies, however, suggest that both cerebellar hemispheres are recruited during early online learning independent from the hand used for task execution [2, 22, 23]. Finally, Seidler and colleagues [24] reported no association of cerebellar activation with initial motor sequence learning, while they found significant cerebellar activation during reproduction of the previously learned sequence. Therefore the possibility remains that—although the majority of the evidence discussed above supports the view of a relevant role of the cerebellum for motor sequence learning—the cerebellum may not be directly involved in the process of motor learning but may actually only be associated with modification of motor performance.

Since neuroimaging and brain stimulation studies have not provided an unambiguous picture of the role of the cerebellum in motor sequence learning, we took a different approach. In the current behavioural study, we investigated online motor sequence learning and offline consolidation of training-induced skill formation in subjects with chronic cerebellar lesions due to ischemic stroke and in healthy controls. We chose to investigate this collective to avoid potential confounds of task performance by ongoing spontaneous recovery processes in the acute or subacute phase after stroke. The results will provide insights into the significance of impaired structural integrity of the cerebellum for motor sequence performance, online motor learning, and offline consolidation.

## Methods

### Participants

Twenty-four participants with chronic unilateral or bilateral cerebellar lesions due to stroke between the age of 18 and 80 years were recruited. All of these participants had been treated on the stroke unit of the Department of Neurology at the University of Leipzig at the time of the acute stroke. Additionally, 25 age- and sex-matched healthy control participants were recruited from Leipzig and the surrounding area. According to the Edinburgh Handedness Inventory [25], all participants were right-handed except one patient with chronic cerebellar stroke and two control participants who were left-handed. To ensure that dexterous finger movements similar to the task employed in our study were not “overlearned,” professional musicians and professional typists were excluded. Both chronic cerebellar stroke patients and control participants were naive to the employed motor sequence learning task. None of the participants reported a history of sleep disorders, previous stroke, or serious medical, neurological or psychiatric diseases. Additionally, all participants received a neurological examination to screen for stroke-related and other neurological deficits. At the time of the study, none of the patients showed neurological impairments that could relevantly interfere with performing the motor sequence learning task. Participants showed no signs of cognitive impairment as assessed by the Mini-Mental State Examination [26] (exclusion cut-off < 26) and no relevant depressive symptoms according to the short version of the Beck Depression Inventory (BDI) [27] (exclusion cut-off > 19). Data from one patient and data from two control participants were excluded from further analysis due to poor motor sequence training performance (i.e., task performance was > 2 standard deviations lower than average group performance), leaving data from 23 cerebellar stroke patients and 23 control subjects for further analysis (please see Table 1 for detailed demographic information).

**Table 1:** Demographic characteristics of cerebellar stroke patients and healthy controls

	Sex (m/f)	Age (y)	MMSE (score)	BDI (score)	SSS (score)			
					T1	R1	T2	R2
Cerebellar stroke patients	16/7	60.0 ± 15.6	28.4 ± 1.2	7.7 ± 5.3	1.7 ± 0.6	1.7 ± 0.6	1.6 ± 0.5	1.6 ± 0.6
Control group	15/8	57.2 ± 18.9	28.8 ± 1.1	4.4 ± 3.1	1.6 ± 0.5	1.6 ± 0.6	1.4 ± 0.5	1.5 ± 0.6
Group difference (*p* values)	0.760	0.580	0.260	0.014	0.411	0.335	0.080	0.456

Displayed are mean values ± standard deviation

*Abbreviations*: *m* male, *f* female, *y* years, *MMSE* Mini Mental State Examination, *BDI* Beck Depression Inventory, *SSS* Stanford Sleepiness Scale, *T* Training (Session 1 & 2), *R* Retest (Session 1 & 2)

### Experimental Design

All participants took part in two experimental sessions each corresponding to performing the motor sequence learning task with either one of both hands. Each session encompassed two parts, a training part in the morning at 11 am and a delayed retest part 8 h later (7 pm) to assess consolidation of training-induced task skill acquisition. Sessions were separated by at least 14 days to prevent carry-over effects. In patients with unilateral cerebellar lesion, the order of sessions was balanced with respect to practicing with the ipsilesional or contralesional hand. In healthy controls and patients with bilateral lesions, the order of sessions was counterbalanced with respect to practicing with the dominant or non-dominant hand. Before each training and each retest, alertness of participants was assessed with the Stanford Sleepiness Scale [28].

### Motor Sequence Learning Task

**Fig. 1:**
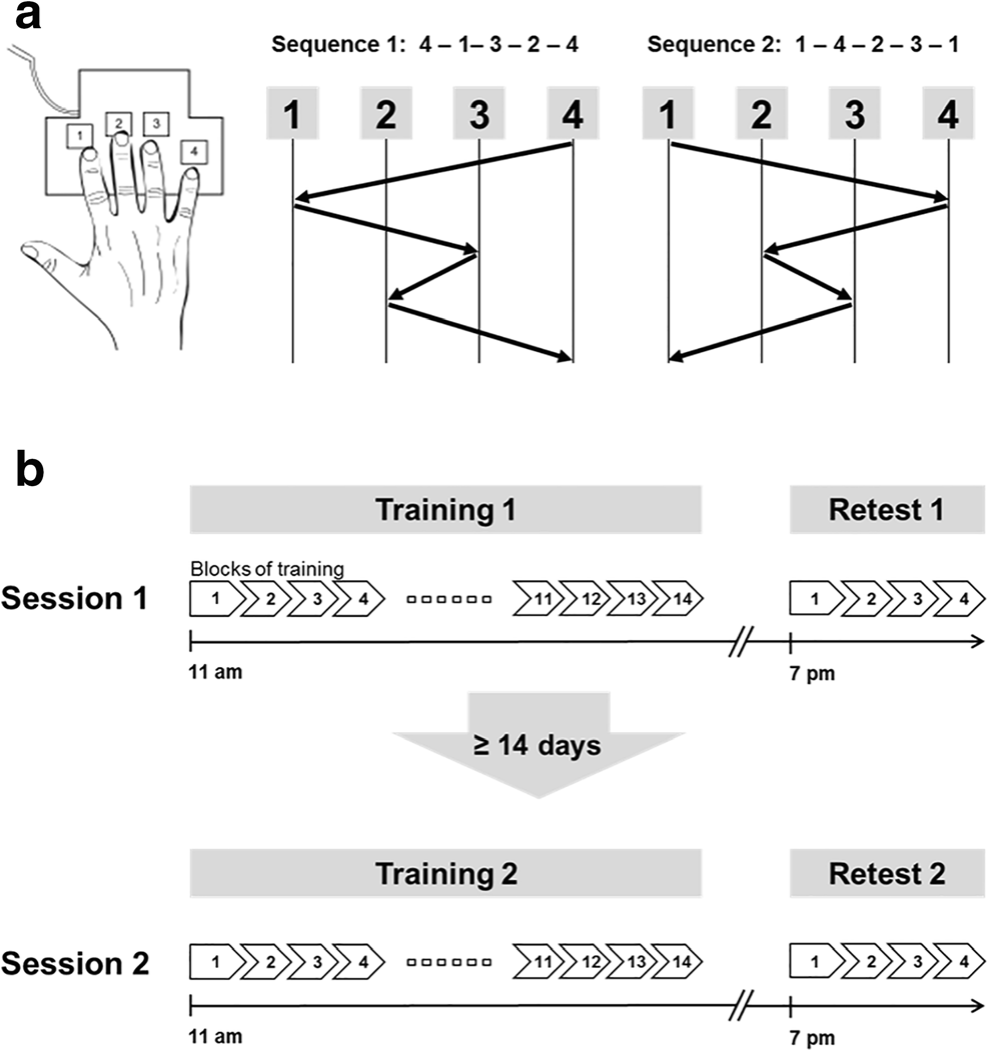
Experimental design. **a** Finger positioning on the keyboard and sequence order exemplary for the right hand. **b** The experiment consisted of two sessions which were separated by at least 14 days. Each session contained a training phase and a delayed retest phase. Training (11 am) encompassed 14 task blocks of 60 key presses and 25-s rest periods in-between blocks. Delayed retesting took place 8 h later (7 pm) and encompassed 4 blocks of the task

Motor sequence performance was assessed by an adapted version of the sequential finger tapping task introduced by Karni and colleagues [4], which has also been employed extensively in our laboratory [29–31]. In each of both experimental sessions, participants were asked to practice one of two different, equally difficult five-element finger-tapping sequences on a four-button customized keyboard. Sequence 1 was “4-1-3-2-4” and sequence 2 was “1-4-2-3-1” (1 = index finger, 2 = middle finger, 3 = ring finger, 4 = little finger). The order of type of sequence allocation to session 1 or session 2 was balanced across all participants. To verify that participants had explicit knowledge of the finger-tapping sequence prior to training onset, they were required to slowly repeat the sequence until they reproduced it three times in a row without mistakes. The training part encompassed 14 successive blocks of sequence execution which were separated by 25-s rest periods. Participants were instructed to perform the sequence as fast as possible while making as few errors as possible. Unbeknownst to the participants, each task block was terminated after 60 key presses to make sure that all participants performed the same number of training movements (i.e., all participants received the same amount of training). Therefore, a maximum of 12 correct sequences could be executed within each block of training. The beginning of a training block was indicated by a green fixation cross in the middle of a computer screen in front of the participants, which turned red (i.e., after 60 button presses on the keyboard) to indicate the beginning of a 25-s rest block. The delayed retest session after an interval of 8 h encompassed only 4 blocks of the task. During training and retesting no information on the sequence was presented to the participants (Fig. 1).

### Data Acquisition and Analysis

Task performance was recorded with a customized four-button keyboard and processed using customized Matlab (Mathworks, Natick, USA) scripts to extract speed and accuracy of sequence execution. Speed of sequence execution was defined as the average time needed to complete correct sequences within each task block (time to perform a correct sequence, TCS). Accuracy was defined as the number of correct sequences in a given block. Accordingly, errors were defined as the maximum number of correct sequences per block (i.e., 12) minus the actual number of correct sequences within each block. To take into account interindividual differences with respect to the strategy to improve task performance (e.g., focus on speed performance at the expense of accuracy), task performance was quantified using a performance index (PI) that incorporates speed performance and accuracy according to the following formula [32, 33]:

PI_*x*_ *=* exp − (TCS_*x*_) *×* exp − (errors_*x*_/12) *×* 100, where *x =* block of trials*.*

Effects on task performance were assessed by applying a repeated measures analysis of variance (rmANOVA) to the training PI values with group as the between-subjects factor and block and session (e.g., dominant vs. non-dominant hand, or ipsilesional vs. contralesional as applicable) as within-subject factors. This allowed us to investigate performance changes as a function of training (main effect of block), for differences in the rate of learning with respect to the factor hand (block * hand interaction), for general task performance differences during training between sessions (e.g., between training with the contralesional and ipsilesional hand) and for differences of task performance between chronic cerebellar stroke patients and healthy controls.

To quantify consolidation, offline changes of PI measures were assessed between the individual end-of-training performance (EOT; i.e., average PI of the last 4 blocks of training) and the first block of delayed retesting according to the following formula:

$$ \mathrm{normalized}\ \mathrm{PIretest}\_\mathrm{block}1=\left(\mathrm{PIretest}\_\mathrm{block}1-\mathrm{PIEOT}\right)/\mathrm{PIEOT} $$

Therefore, positive normalized PI values (reported as percentage) indicate gains of task performance relative to the individual end-of-training performance, while negative normalized PI values indicate relative performance decrements. We chose to use only the first block of retesting to compute consolidation to avoid contamination of the consolidation measure with additional online learning. rmANOVA of normalized PI values with factors group, block, and session allowed us to test for differences of consolidation with respect to group allocation or with respect to the hand (e.g., ipsilesional vs. contralesional) used for task performance. This procedure also provided information on performance changes driven by additional task training during the retest (main effect of block), and on potential differences with respect to learning rate during the retest (group × block × session interaction).

Spearman’s rank correlation coefficient was applied to assess associations of motor performance and consolidation with age, lesion volume and interval since stroke onset.

For all statistical tests, the alpha level was set to *p* < 0.05. rmANOVAs were checked for violation of sphericity and degrees of freedom and *p* values were corrected if necessary (Greenhouse-Geisser). All statistical analyses were carried out with SPSS 24 (SPSS, Chicago, IL, USA).

Cerebellar stroke lesion location and distribution were assessed based on MRI and CT images acquired for clinical purposes during the acute phase of the cerebellar stroke. Individual FLAIR-weighted images were normalized to Montreal Neurological Institute (MNI) space in SPM 12 (https://www.fil.ion.ucl.ac.uk/spm/software/). Anatomical labelling of damage to specific cerebellar structures was based on automated anatomical labelling [34] distributed with MRIcron (https://www.nitrc.org/projects/mricron) and the SUIT atlas [35, 36].

## Results

**Fig. 2:**
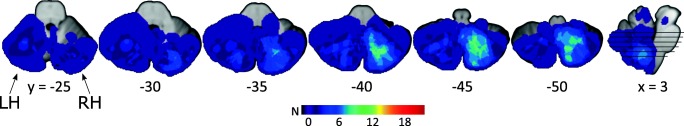
Visualization of lesion distribution. Colorbar specifies number of patients (*n* = 23) with overlapping lesions in each voxel. Hot colors indicate a greater number of patients with lesions in the respective region. Maximum lesion overlap (*n* = 12) was found in the right postero-lateral cerebellum corresonding to Crus II (MNI coordinates 20, − 69, − 40). For this representation, individual FLAIR-weighted images were normalized to Montreal Neurological Institute (MNI) space in SPM 12 (https://www.fil.ion.ucl.ac.uk/spm/software/). Lesions were manually delineated and superimposed on the SUIT template using MRIcron (https://www.nitrc.org/projects/mricron). RH, right cerebellar hemisphere; LH, left cerebellar hemisphere

Demographic baseline characteristics (Table 1) were similar in healthy controls and cerebellar stroke patients except for a slightly higher BDI score in the patient group. However, mean BDI scores in patients were, similar to healthy controls, below the cut-off for even a mild depressive syndrome. Detailed information on lesion characteristics in the cerebellar stroke patients is given in Table 2 and Fig. 2. Out of 23 cerebellar stroke patients, 18 had suffered from unilateral cerebellar stroke (7 in the left and 11 in the right cerebellar hemisphere) and 5 patients had suffered from bilateral cerebellar stroke.

**Table 2:** Detailed patient and cerebellar lesion characteristics

Cerebellar stroke patients										Control group		
No.	Age (y)	Sex (m/f)	ISS (months)	Affected hemisphere	Vascular territory	Affected cerebellar structures				No	Age (y)	Sex (m/f)
						Nuclei	Vermis	Hemisphere	Volume (cm^3^)			
1	77	F	7	Right	PICA	n.a.	n.a.	r: CRII, VIIb, VIII	0.1	1	68	f
2	78	M	7	Left	PICA	r: DN	n.a.	l: CRI, CRII, VIIb, VIII, IX	11.4	2	80	m
3	39	M	35	Right	PICA	r: DN	n.a.	r: CRI, IV-VI, VIII, IX	2.5	3	38	m
4	34	M	3	Right	PICA	r: DN, FN, IN	I-X	r: CRI, CRII, III-VI, VIIb, VIII, IX	10.3	4	23	m
5	74	M	25	Bilateral	SCA	b: DN, FN, IN	I-X	r: CRI, CRII, VI, VIIb, VIII-X l: CRI, CRII, III-VI, VIIb, VIII-X	73.7	5	74	m
6	75	M	25	Bilateral	PICA	b: DN	VIII-X	r: CRI, CRII, VIIb, VIII, IX l: CRI, CRII, VIIb, VIII, IX,	66.1	6	74	m
7	66	F	7	Right	SCA	r: DN, IN	VII-X	r: CRI, CRII, VI, VIIb, VIII, IX	34.3	7	71	f
8	80	F	11	Bilateral	PICA SCA	l: DN, FN, IN	I-IX	r: CRI, CRII, III-VI, VIIb, VIII, IX l: CRI, CRII, III-VI, VIIb, VIII, IX	54.8	8	69	f
9	56	M	17	Right	PICA	n.a.	n.a.	r: VIIb, VIII, IX	5.9	9	55	m
10	62	M	16	Right	PICA	n.a.	n.a.	r: CRI, CRII, VIIb, VIII	17.0	10	67	m
11	75	M	10	Bilateral	PICA	b: DN r: IN	VI-IX	r: CRI. CRII, VI, VIIb, VIII- X l: CRI, CRII, VIIb, VIII, IX,	75.7	11	76	m
12	57	M	3	Right	PICA	r: DN, IN	VIII-IX	r: CRI, CRII, VI, VIIb. VIII, IX	15.8	12	57	m
13	77	M	12	Right	PICA	r: DN	VIII	r: CRI, CRII, VI, VIIb, VIII, IX	4.3	13	78	m
14	72	M	12	Left	SCA	l: DN, IN	III-VI	l: CRI, III, IV, V, VI	3.7	14	69	m
15	55	M	16	Left	PICA	n.a.	n.a.	l: CRII, VIIb, VIII	2.0	15	53	f
16	28	F	32	Left	PICA	n.a.	VIII	l: CRI, CRII, VIIb, VIII	2.5	16	19	f
17	65	M	9	Left	PICA	n.a.	n.a.	l: CRI, CRII, VIIb, VIII	0.2	17	67	m
18	42	M	9	Right	PICA	l: DN	VIII-X	r: CRI, CRII, VI, VIIb, VIII, IX	26.2	18	41	m
19	45	F	14	Left	PICA	l: DN	IX-X	l: VIII, IX	15.7	19	36	f
20	50	F	25	Right	PICA	r: DN	VIII	r: CRI, CRII, VIIb, VIII-X	22.7	20	40	f
21	43	F	33	Bilateral	PICA	r: DN	n.a.	r: CRI, CRII; VI, VIII l: CRI, CRII, VI, VIIb, VIII	8.2	21	28	f
22	75	M	28	Left	SCA	n.a	n.a.	l: CRI, CRII, VI, VIIb, VIII	3.1	22	76	m
23	56	M	3	Right	PICA	r: DN	VIII-IX	r: CRI, CRII, VIIb, VIII, IX	19.9	23	56	m

*Abbreviations*: *y* years, *ISS* interval since stroke onset, *m* male, *f* female, *PICA* Posterior inferior cerebellar artery, *SCA* superior cerebellar artery, *DN* dentate nucleus, *IN* interposed nucleus, *FN* fastigial nucleus, *CRI* Crus I, *CRII* Crus II, *n.a.* not affected, *Volume* lesion volume in cm^3^, *b* bilateral, *r* right, *l* left

### Motor Sequence Performance Is Impaired in Chronic Cerebellar Stroke Patients While Online Motor Sequence Learning Remains Intact

**Fig. 3:**
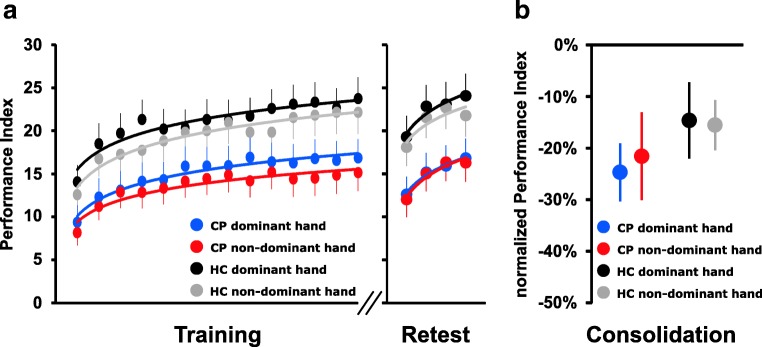
**a** Task performance. Mean performance index (PI) values across the 14 blocks of training and the 4 blocks of delayed retesting for task execution with the dominant and the non-dominant hand in chronic cerebellar stroke patients (CP) and healthy control participants (HC). Vertical bars represent the standard error of the mean (SEM). (B) Consolidation. Columns represent mean normalized PIs of the first block of retesting for cerebellar patients (CP) and controls (HC). Positive values would indicate performance increments while negative values indicate performance decrements relative to the individual end-of-training performance baseline (average performance across the last 4 blocks of training). Bars represent SEM

As detailed below, there was no difference in task performance with the ipsilesional or contralesional hand among patients with unilateral cerebellar stroke lesions (please see separate subgroup analyses). This allowed us to perform a rmANOVA conducted on the PI values of the 14 training blocks with *group* (patients vs. controls) as between-subject factor and *block* and *session* (dominant vs. non-dominant hand) as the within-subject factors, without further separation of contralesional and ipsilesional hand in patients with unilateral cerebellar stroke lesions. This rmANOVA revealed a significant main effect of *group* (*F*_(1,44)_ = 4.096, *p* = 0.049), which was driven by a lower average PI across both training sessions in patients (14.3, 95% confidence interval (CI) 10.2–18.4) compared to healthy controls (20.2, CI 16.1–24.3). Importantly, rmANOVA demonstrated a significant main effect of *block* (*F*_(5.047,222.069)_ = 48.250, *p* < 0.001) and *session* (*F*_(1,44)_ = 4.699, *p* = 0.036) but no significant interaction of *group × block* (*F*_(5.047,222.069)_ = 1.403, *p* = 0.224), *group × session* (*F*_(1,44)_ = 0.009, *p* = 0.924), nor a significant *group × block × session* interaction (*F*_(6.439,283.310)_ = 1.075, *p* = 0.378). Average PI in the first block of both training sessions was 8.8 (CI 5.1–12.4) in patients and 13.3 (CI 9.7–17.0) in healthy controls and increased to 16.0 (CI 11.5–20.5) and 23.0 (CI 18.4–27.5), respectively, for average performance during the last block of both training sessions (Fig. 3a). The significant main effect for the factor *session* was driven by larger average PI values in the training session with the dominant hand (18.0, CI 15.0–21.1) compared to when training was performed with the non-dominant hand (16.5, CI 13.5–19.4). Collectively, these results indicate that, although patients with a chronic cerebellar stroke lesion demonstrated a lower overall motor sequence performance, there were no relevant differences between patients and healthy controls with respect to the rate of training-induced performance increments (i.e., online learning) irrespective of the hand (dominant vs. non-dominant) used for training.

Similar results were obtained, when training performance was analyzed only for the very early training phase (blocks 1–4) during which performance increases rapidly as a function of practice. We additionally performed this analysis as previous research proposed an important role of the cerebellum primarily during early online learning before training-induced performance increments level-off with ongoing training (e.g., [9, 12]). A rmANOVA including only the PI values of the first 4 blocks of training showed a significant main effect of *group* (*F*_(1,44)_ = 4.083, *p* = 0.049), *block* (*F*_(2.196,96.626)_ = 56.482, *p* < 0.001), and *session* (*F*_(1,44)_ = 4.256, *p* = 0.045), while no significant interaction of any of the factors was observed (all *p* ≥ 0.344). These results obtained for the very early training phase further support that, despite impaired motor performance, chronic cerebellar stroke patients did not differ from healthy controls with respect to speed and magnitude of training-induced performance increments.

Training-induced performance increments levelled off at the end of the training sessions in patients and controls as indicated by a non-significant effect of *block* (*F*_(2.450,107.804)_ = 1.335, *p* = 0.268) and the absence of a significant interaction of factors *group*, *block*, and *session* (all *p* ≥ 0.622) when running the rmANOVA across the last four blocks of training. This verifies that patients and controls reached asymptotic performance levels at the end of both training sessions. Therefore, average performance across the last four blocks of training was considered as the “end-of-training-baseline” (EoT) against which potential consolidation effects were assessed.

Correlation analysis revealed a highly significant negative association of age and training performance (average PI across both trainings) in both healthy controls (rho = −0.643, *p* = 0.001) and in patients (rho = −0.690, *p* < 0.001). Of note, no significant correlation was found in patients between average training performance and the interval since stroke onset (months, rho = 0.269, *p* = 0.214) nor for the volume of the cerebellar stroke lesion (cm^3^, rho = −0.178, *p* = 0.417).

### No Impairment of Motor Sequence Consolidation in Chronic Cerebellar Stroke Patients

Driven by a lower average PI across retests in patients (15.0, CI 10.5–19.6) compared to healthy controls (21.7, CI 17.1–26.2), rmANOVA conducted on the PI values of the 4 retest blocks revealed a significant main effect of *group* (*F*_(1,44)_ = 4.354, *p* = 0.043) indicating a persisting relative impairment of motor performance in patients compared to the control group during delayed retesting. Moreover, rmANOVA demonstrated a significant effect of *block* (*F*_(2.137,94.026)_ = 52.402, *p* < 0.001) in the absence of a significant effect of *session* (*F*_(1,44)_ = 1.769, *p* = 0.190) or a significant interaction of any of the factors (all *p* ≥ 0.313; Fig. 3a). In sum and congruent with the previous analysis of the training performance, results support the conclusion that groups did not differ with respect to their online learning abilities despite persisting impaired motor sequence task performance in patients during retesting.

Consolidation was assessed by normalizing the PI values of the first block of delayed retesting to the individual EoT performance baseline of the corresponding training session. Mean normalized retest PI of the first block of retesting amounted to − 15.1% (CI − 25.0 to − 5.2) in chronic cerebellar stroke patients and − 23.1% (CI − 33.0 to − 13.3) in healthy controls. A rmANOVA with factors *group* and *session* (dominant hand vs. non-dominant hand) conducted on the normalized PI values revealed no significant main effect for *group* (*F*_(1,44)_ = 1.346, *p* = 0.252) and *session* (*F*_(1,44)_ = 0.028, *p* = 0.867), and no significant interaction of both factors (*F*_(1,44)_ = 0.094, *p* = 0.761; Fig. 3b). Of note, we had chosen to assess consolidation by normalizing performance during only the first block of delayed retesting to the individual end-of-training performance to avoid contamination of the offline consolidation measure by additional online learning during retesting. However, results remain qualitatively similar if consolidation is assessed across all 4 blocks of retesting (main effect of *group:* (*F*_(1,44)_ = 0.060, *p* = 0.808), *session*: *F*_(1,44)_ = 0.980, *p* = 0.328), *group* × *session* interaction: *F*_(1,44)_ = 1.372, *p* = 0.248).

Correlation analysis revealed a significant negative association of age and consolidation (mean normalized PI of both sessions) across patients and healthy controls (rho = − 0.454, *p* < 0.002). Notably, there was no relevant association of consolidation with lesion volume (cm^3^, rho = − 0.094, *p* = 0.670) nor with the interval since stroke onset (months, rho = 0.298, *p* = 0.167) in the patients group.

Collectively, while the relative deficit of chronic cerebellar stroke patients in performing the sequential motor task persisted during delayed retesting, findings suggest similar capabilities of patients and controls to consolidate training-induced motor skill.

### No Difference Between Task Execution with the Ipsilesional or Contralesional Hand with Respect to Online Learning Or Consolidation in Chronic Cerebellar Stroke Patients

In an additional analysis, we further explored whether training with either the ipsilesional or contralesional hand with respect to the cerebellar lesion had an effect on online learning or consolidation within the patient group. To this end, we excluded those patients from the analysis whom had suffered from bilateral cerebellar stroke (*n* = 5) and analyzed training and retest performance in the remaining 18 patients (7 patients with lesions in the left cerebellar hemisphere, 11 patients with lesions in the right cerebellar hemisphere).

**Fig. 4:**
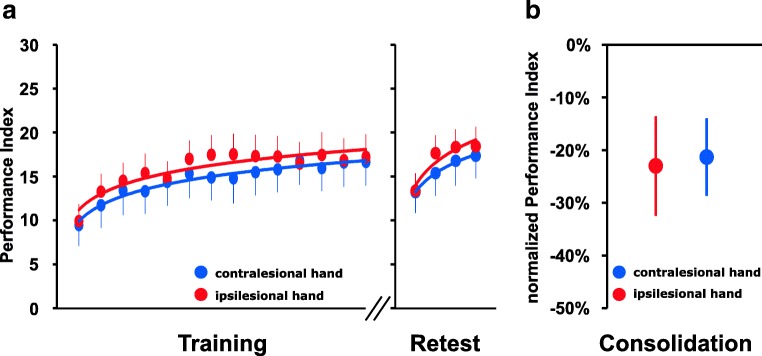
**a** Task performance. Mean performance index (PI) values across the 14 blocks of training and the 4 blocks of delayed retesting for task execution with the ipsilesional and the contralesional hand in patients with a unilateral chronic cerebellar stroke lesion. Vertical bars represent the standard error of the mean (SEM). **b** Consolidation. Columns represent mean normalized PIs of the first block of retesting for task execution with the ipsilesional and the contralesional hand in patients with a unilateral chronic cerebellar stroke lesion. Positive values would indicate offline performance increments while negative values indicate performance decrements relative to the individual end-of-training performance baseline (average performance across the last 4 blocks of training). Bars represent SEM

A rmANOVA conducted on the PI values across both training sessions with the within subject factors block and session (training with the ipsilesional vs. contralesional hand) revealed a significant effect of *block* (*F*_(3.958,67.283)_ = 18.517, *p* < 0.001) but no significant effect of *session* ( F[1, 17]=1.626, *p* = 0.219) nor a significant interaction of *block* × *session* (*F*_(3.176,53.992)_ = 1.213, *p* = 0.315). Furthermore, we found no significant association of training performance and lesion volume when correlations were calculated separately for mean training performance with the ipsilesional hand (rho = − 0.065, *p* = 0.798) and the contralesional hand (rho = − 0.061, *p* = 0.810). This indicates similar performance as well as similar magnitude and speed of learning independent of whether the task was executed with the ipsilesional or contralesional hand (Fig. 4a).

Similar results were obtained for the analysis of retest performance as rmANOVA across retest PIs revealed a significant effect of *block* (*F*_(3,51)_ = 30.671, *p* < 0.001), but again no significant effect of *session* (*F*_[1, 17]_=1.565, *p* = 0.228) nor a significant interaction of both factors (*F*_(2.249,38.238)_ = 1.956, *p* = 0.151; Fig. 4a).

Finally, we tested for potential differences of consolidation following task training with the ipsilesional and contralesional hand. Mean normalized retest PI of the first block of retesting amounted to − 23.0% (CI − 43.0 to − 3.1) when task training was executed with the ipsilesional hand and − 21.3% (CI − 36.9 to − 5.8) when the contralesional hand was used for task execution. Moreover, correlation analysis revealed no significant association of lesion volume and consolidation when analyzed separately in terms of consolidation following training with the ipsilesional (rho = − 0.168, *p* = 0.505) or contralesional hand (rho = 0.158, *p* = 0.531). Accordingly, rmANOVA across the normalized PI values of the first block of delayed retesting revealed no significant main effect of *session* (*F*_(1, 17)_ = 0.017, *p* = 0.899) indicating similar consolidation of training-induced performance increments when the task was performed with the ipsilesional or contralesional hand (Fig. 4b). Again, results remained qualitatively similar if consolidation was assessed across all 4 blocks of retesting (main effect of *session*: *F*_(1, 17)_ = 0.505, *p* = 0.487), *block* × *session* interaction: *F*_(3,51)_ = 2.079, *p* = 0.115). Overall, the additional exploratory analysis above revealed no evidence of lateralized cerebellar functions with respect to online learning or consolidation of training-induced motor sequence skill.

## Discussion

The present study demonstrates that chronic cerebellar stroke lesions are associated with a persisting impairment of motor sequence execution while neither online skill acquisition during training nor post-training offline consolidation are altered in comparison with a healthy control group. Of note, although unilateral cerebellar lesions often lead to severe motor function impairments ipsilateral to the lesioned cerebellar hemisphere in the acute phase, our findings acquired in chronic cerebellar stroke patients were independent of whether the explicit motor sequence learning task was executed with the ipsilesional or contralesional hand. Collectively, present findings point to an essential, non-lateralized function of the cerebellum for motor control that cannot be compensated in case of cerebellar damage, whereas neither online motor learning nor offline motor consolidation were relevantly affected in chronic cerebellar stroke.

Our results are in line with and extend findings from a study in a smaller sample of chronic cerebellar stroke patients by Dirnberger and colleagues [37], who demonstrated impaired motor sequence performance during execution of an implicit serial reaction time task while within-session performance increments induced by repeated sequence execution were comparable to those of healthy controls. Additional evidence for the interpretation that the cerebellum has a primary function in modification of motor sequence performance but not motor sequence learning can be derived from a functional magnetic resonance imaging study of Seidler and colleagues [24] who found no association of cerebellar activation with initial motor sequence learning, while there was significant cerebellar activation during reproduction of the previously learned sequence. However, current results, which indicate no impairment of online motor learning in cerebellar stroke patients, may seem surprising, given the large body of evidence that suggests a prominent role of the cerebellum during early online motor learning [3, 8, 9, 12, 13]. This discrepancy may be resolved by the fact that cerebellar stroke patients were investigated in the chronic phase of the disease and potential initial impairments of cerebellar function for motor learning may have been compensated despite a persisting deficit of motor sequence performance. Others, however, reported an impairment of motor sequence learning in patients with chronic unilateral cerebellar stroke lesions, regardless of the hand used (ipsilesional or contralesional) for task execution [23], and in patients suffering from chronic cerebellar degeneration [38]. Gomez-Beladarrain and colleagues [21] even proposed selective impairment of procedural online learning with the hand ipsilateral to the unilateral cerebellar stroke lesion, while learning with the contralesional hand was intact. Lateralized functions of the cerebellum with respect to motor learning may also be supported by findings of Gheysen and colleagues [14], who demonstrated bidirectional modulation of motor sequence learning by unilateral “inhibitory” or “facilitatory” priming of the cerebellum by use of transcranial theta burst stimulation in young and healthy subjects. However, in contrast to the findings in patients with unilateral cerebellar stroke [21], motor learning in healthy subjects was only modulated when the cerebellar hemisphere contralateral (but not ipsilateral) to the hand that executed the task was targeted by stimulation. In our sample of chronic cerebellar stroke patients, motor performance and motor learning were similar, independent of whether the motor learning task was executed with the ipsilesional or contralesional hand. Therefore, current results do neither support the interpretation of lateralized functions of the cerebellum during motor sequence performance nor for online motor learning, which is in line with previous research demonstrating bilateral activation of the cerebellum during sequence execution and motor learning independent of the hand used for training [2, 10, 22]. Yet, we cannot exclude the presence of lateralized impairments of motor sequence performance or motor sequence learning in the acute phase after cerebellar stroke. However, we had decided to investigate patients in the chronic stage after cerebellar stroke as at this point it can be assumed that spontaneous recovery is completed and that stroke-related persisting functional deficits can be considered stable. Thereby, we avoided potential confounds of motor performance, motor learning and motor consolidation by intra- and inter-subject differences in terms of the dynamics of ongoing spontaneous recovery in the acute and subacute phase after stroke. Because all of our cerebellar stroke patients were in the chronic stage of the disease (average interval since stroke onset 16 months) and no association of the interval since stroke onset with motor performance was revealed, we can assume that initial stroke-related deficits of all non-essential functions of the cerebellum for motor-learning—if there had been any in the acute phase—were compensated by the time of the experiment. Collectively, our results may suggest that cerebellar function is negligible for online motor sequence learning. Alternatively, potential initial stroke-related impairment of cerebellar function with respect to motor learning is accessible to sufficient compensation by local or remote mechanisms. The function of the cerebellum for motor sequence performance, however, seems to be an essential cerebellar function that cannot be completely compensated.

One may ask whether the discrepancy between the current results that indicate no relevant role of the cerebellum during online motor sequence learning and previous findings that indicated impaired online motor learning in chronic cerebellar stroke patients [21, 23] and patients with degenerative cerebellar diseases [38] may be explained by the nature of the employed tasks. While an explicit motor sequence learning task was used in the current study, almost all previous studies mentioned above employed implicit serial reaction time tasks. Although cerebellar activation has been seen in implicit as well as in explicit motor sequence learning, both types of tasks are differently related to other domains that have also been implicated in motor sequence learning and are associated with cerebellar activation (for review see [13]). For example, in implicit motor sequence learning both visuospatial working memory and verbal working memory are correlated with motor sequence learning [39, 40], while explicit motor sequence learning has been only associated with visuospatial working memory [41, 42]. Differences in task demands with respect to cerebellar recruitment during explicit and implicit sequence learning may, therefore, partly explain the contrasting results of the current study and previous results. However, cerebellar areas activated during explicit and implicit motor sequence learning overlap [13] and, more importantly, the one study that demonstrated qualitatively similar results in cerebellar stroke patients as our study (i.e., impairment of sequence performance but not online learning, [37]) employed an implicit, but not explicit, serial reaction time task. It is, therefore, unlikely that the contrasting results can solely be attributed to differential cerebellar recruitment in explicit and implicit motor sequence learning tasks. Another reason for the differing results may be related to the investigated sample sizes. Although—to our best knowledge—the current study investigated motor sequence learning in the largest sample (*n* = 23) of cerebellar patients so far, diverging results in-between previous studies investigating implicit learning as well as differences with respect to the current study may be related to the relatively small sample sizes of investigated patients in the aforementioned studies (*n* = 8 in [23], *n* = 11 in [37], *n* = 15 in [38], *n* = 14 in [21]).

Besides investigating the impact of chronic cerebellar stroke lesions on online motor sequence learning, our study also advances the existing body of knowledge on the role of the cerebellum in post-training offline consolidation. Our results indicate similar capacity to consolidate training-induced skill formation in chronic cerebellar stroke patients and healthy controls despite the persisting impairment of motor sequence execution also during delayed retesting in patients. Moreover, and similar to the findings for the online learning capacity of patients, no association of consolidation with the interval since stroke onset or the volume of the cerebellar stroke lesion was detected. Furthermore, consolidation did not differ dependent on whether the ipsilesional or contralesional hand was used for training and delayed retesting. Of note and concurring with the results from the training session, patients and healthy controls also demonstrated no difference with respect to online learning during delayed retesting. These findings, again, indicate an essential role of the cerebellum for motor sequence execution, while no indication of a relevant role of the cerebellum for online learning or offline skill consolidation can be derived from our data. Our findings and interpretation with respect to the role of the cerebellum for consolidation are in line with the important theoretical models already discussed above that proposed no relevant role of the cerebellum for offline consolidation as cerebellar activity was shown to diminish across training to become untraceable when training-induced skill increments level off [3, 5, 9, 12, 13]. This view may be challenged by findings of Wessel and colleagues [19] who demonstrated specific facilitation of post-training offline consolidation of training-induced motor skill formation in the absence of an effect on online learning, when training was combined with concurrent anodal cerebellar transcranial direct current stimulation (tDCS). Others, however, demonstrated opposite results when motor sequence learning was combined with anodal tDCS, i.e., cerebellar tDCS specifically facilitated online learning while consolidation remained unaffected [16]. Although our study provides no indication of a relevant role of the cerebellum in consolidation, we, again, cannot rule out the possibility of initial deficits of consolidation in the acute phase of the cerebellar stroke that may have been compensated by effective local or remote brain plasticity by the time of the investigation.

Compared to non-invasive brain stimulation and functional neuroimaging studies, the lesion approach may be a valuable complementary method with regard to the identification of the causal role of a brain structure, i.e., the cerebellum in the current study. Both approaches have their specific limitations: In the case of non-invasive brain stimulation, uncertainty remains regarding physiological effects on the target structure, whereas in chronic lesions, the studied structure may be functionally compensated by the unaffected brain regions. The present findings fail to provide evidence of involvement of the cerebellum in online motor sequence learning or offline consolidation. If these findings are not the consequence of functional compensation, but instead reflect cerebellar absence in these stages of motor sequence learning, then it may perhaps explain the inconsistent results of the cerebellar stimulation studies.

## Conclusions

Our results suggest that motor sequence performance is persistently impaired in patients with chronic cerebellar stroke lesions, independent of whether the task is executed with the ipsilesional or contralesional hand. However, neither the capacity to improve sequential motor skill as a function of training nor the capacity to consolidate training-induced skill formation was altered in comparison with healthy control subjects. These findings point to an essential function of the cerebellum for motor sequence performance that is not completely accessible to compensatory mechanisms. On the other hand, its role for online and offline sequence learning seems to be negligible or, alternatively, accessible to local or remote compensation mechanisms. Findings may have implications for rehabilitative strategies in chronic cerebellar stroke. The fact that the capacity to learn and consolidate new skills was intact implies that persisting functional impairments caused by cerebellar stroke may still be alleviated even months after disease onset by additional skill training.

## Data Availability

The data used to support the findings of this study are available from the corresponding author upon reasonable request.
